# Successful treatment of *Candida parapsilosis *mural endocarditis with combined caspofungin and voriconazole

**DOI:** 10.1186/1471-2334-6-73

**Published:** 2006-04-11

**Authors:** Víctor López-Ciudad, María J Castro-Orjales, Cristóbal León, César Sanz-Rodríguez, María J de la Torre-Fernández, Miguel A Pérez de Juan-Romero, María D Collell-Llach, María D Díaz-López

**Affiliations:** 1 Intensive Care Unit, Hospital Santa María Madre-Complejo Hospitalario de Ourense, Ourense, Spain; 2 Intensive Care Unit and Emergency Service, Hospital Universitario de Valme, Sevilla, Spain; 3 Department of Clinical Research, Merck Sharp & Dohme de España, S.A. Madrid, Spain; 4 Department of Cardiology, Hospital Santa María Madre-Complejo Hospitalario de Ourense, Ourense, Spain; 5 Unit of Infectious Diseases, Hospital Santa María Madre-Complejo Hospitalario de Ourense, Ourense, Spain

## Abstract

**Background:**

Fungal mural endocarditis is a rare entity in which the antemortem diagnosis is seldom made. Seven cases of mural endocarditis caused by *Candida *spp. have been collected from literature and six of these patients died after treatment with amphotericin B.

**Case presentation:**

We report a case of mural endocarditis diagnosed by transesophageal echocardiogram and positive blood cultures to *Candida parapsilosis*. Because blood cultures continued to yield *C. parapsilosis *despite caspofungin monotherapy, treatment with voriconazole was added.

**Conclusion:**

This is the first description of successful treatment of *C. parapsilosis *mural endocarditis with caspofungin and voriconazole.

## Background

Fungal endocarditis has increased in incidence during the last 2 decades. In a review of 152 cases of fungal endocarditis reported between 1995 and 2002 [1], *Candida *species were recovered in 94.1% of yeast infections and *Aspergillus *species in 71.8% of mold infections. Mural endocarditis is inflammation and disruption of the nonvalvular endocardial surface of the cardiac chambers. Its presentation is remarkably similar to that of infective valvular endocarditis. Risk factors for mural endocarditis include chronic debilitating disease, immunosuppression, intravenous drug use, central venous catheterization, prolonged treatment with broad-spectrum antibiotics, and repeated surgery. The pathogenesis of mural involvement has been ascribed to hematogenous seeding or direct extension of myocardial abscesses [1]. Mural endocarditis has been reported in the setting of infected mural thrombi or aneurysms, jet lesions from ventricular septal defects and idiopathic hyperthrophic subaortic stenosis [2]

Kearney et al. [3] collected 52 patients with mural endocarditis in three autopsy studies and five case reports. Most of these cases corresponded to bacterial mural endocarditis. Fungal mural endocarditis is usually diagnosed in immunocompromised hosts or in patients with predisposing underlying conditions [3]. However, the antemortem diagnosis is seldom made. *Aspergillus *mural endocarditis has been diagnosed in small series of patients [4-7] but mural endocarditis caused by *Candida *spp. has been exceptionally reported [8,9]. We present a case of *Candida parapsilosis *mural endocarditis cured by combined treatment with caspofungin and voriconazole.

## Case presentation

A 59-year-old woman had a 13-year history of biopsy proven alcoholic cirrhosis. She was admitted to the hospital because of abdominal pain, nausea, vomiting, and fever. A diagnosis of biliary acute pancreatitis was established. Twenty days after admission, the patient's condition deteriorated. A new dynamic abdominal computed tomography (CT) scan showed an increase of peripancreatic fluid collections and necrosis of approximately 80% of the gland. A CT-guided fine needle biopsy yielded a positive culture for methicillin-sensitive *Staphylococcus aureus *and enterobacteriacea. The patient was treated empirically with piperacillin/tazobactam and received total parenteral nutrition via a subclavian central venous catheter. Surgical treatment consisted of cholecystectomy, sequestrectomy of necrotic pancreatic tissue, and extensive debridement. Antibiotic treatment was continued for 23 more days, although intravenous fluconazole was added and given for 14 days due to oropharyngeal candidiasis.

The clinical course was complicated by a new episode of abdominal pain and a second operation to drain large fluid collections of the retroperitoneal space was performed. Events during the immediate postoperative period were as follows: 1) treatment with meropenem and teicoplanin was started but fever reappeared, 2) ampicillin-sensitive *Enterococcus faecalis*, methicillin-sensitive *S. aureus*, methicillin-resistant *S. epidermidis*, and *Candida parapsilosis *were documented on intraoperative and drainage cultures, 3) peripheral blood cultures were positive for *C. parapsilosis*, 4) a subclavian catheter was removed and another central venous catheter was inserted into a different site, and 5) culture of the subclavian catheter tip was also positive for *C. parapsilosis*. The minimal inhibitory concentrations (MICs) for the *C. parapsilosis *isolate were as follows: flucytosine, 0.125 μg/mL; amphotericin B, 0.25 μg/mL; ketoconazole, 0.25 μg/mL; fluconazole, 4 μg/mL; and voriconazole, 0.06 μg/mL. On the 5th postoperative day, meropenem was discontinued and intravenous fluconazole, 400 mg/12 h, was initiated. The patient's clinical condition improved. Transthoracic echocardiograms performed 24 hours and 14 days after the onset of treatment with fluconazole were unrevealing. Eye funduscopy was repeatedly negative.

**Figure 1:**
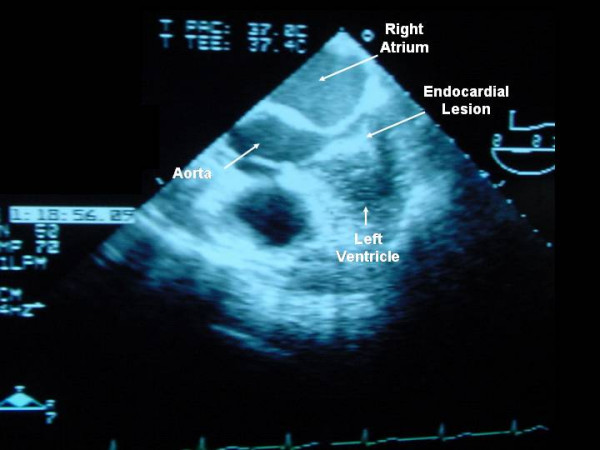
A 2-dimensional transesophageal long-axis echocardiogram showing a mobile, pediculated echodense image adjacent to the lower intraventricular septum.

On the 17th postoperative day, the temperature rose to >38.5°C, the serum C reactive protein concentration increased (>200 mg/L), and peripheral blood cultures grew *C. parapsilosis*. Antifungal treatment with intravenous caspofungin was started (a 70-mg loading dose and then 50 mg daily). A transesophageal echocardiogram showed a mobile, echodense, filamentous image (35 × 10 mm) near the basal part of the interventricular septum, protruding into the left ventricular outflow tract (Figure 1). Myocardial abscesses were not observed. Neither valve involvement nor other intracardiac lesions were observed. Using modified Duke clinical criteria, we diagnosed definite mural endocarditis on the basis of 2 major criteria (repeatedly positive blood cultures for *C. parapsilosis *and an echocardiogram finding positive for endocarditis). Heart surgery was not indicated due to the lack of valvular involvement and the patient's poor clinical condition related to severe pancreatitis, previous abdominal operations, renal dysfunction, and hemodynamic instability. The central venous catheter was replaced and culture of the tip was negative. However, 48 hours after the onset of treatment with caspofungin, serial blood cultures continued to yield *C. parapsilosis*. The serum creatinine clearance was 40 mL/min (normal range 60–120 mL/min) and because of the persistence of fungemia, 72 hours after starting caspofungin therapy, intravenous voriconazole at a dosage of 6 mg/kg/12 h followed by 4 mg/kg/12 h was added. Remarkably, the patient's clinical condition improved and fever subsided. Three days after the combined administration of caspofungin and voriconazole, a negative blood culture was obtained for the first time. Subsequent blood cultures were consistently negative. Serial follow-up transesophageal and transthoracic echocardiograms showed complete resolution of the fungal vegetation without significant aortic valve regurgitation. Treatment with caspofungin was maintained for 60 days. The patient was discharged on oral voriconazole (400 mg/day), which was given for 9 additional months. Oral voriconazole was well tolerated. In November 2005, 16 months after discharge from the hospital, she was asymptomatic without any evidence of infection.

This patient with *C. parapsilosis *mural endocarditis was cured by combined treatment with caspofungin and voriconazole. The outcomes for patients with fungal mural endocarditis have been extremely poor [8]. In the 7 cases of mural endocarditis caused by *Candida* spp. previously reported in the literature, 6 patients died after treatment with amphotericin B [8-10] and the only survivor was treated with combined azole therapy (alternating miconazole and fluconazole) and excision of the two mobile pedunculated masses in the right ventricle [11]. These patients had a mean age of 31 years. Risk factors for mural endocarditis included acute myelocytic leukemia in 5 patients, immunosuppressive treatment for systemic lupus erythematosus in 1, and major abdominal surgery with total parenteral nutrition in 1. The lesion was located in the right ventricle in 6 patients and in the left ventricle in the remaining case. Microbiologic investigation yielded *Candida albicans *in 2 cases, and *C. parapsilosis*, *Candida krusei *and *Candida tropicalis *in one case each; in 2 patients, the species of *Candida *was not identified. Prolonged use of central venous catheter was probably the implicated cause of hematogenous candidiasis and seeding of the endocardium. Intracardiac catheters can produce endothelial damage and predispose usually right-sided endocarditis. The mechanism of direct traumatization of the endocardium by the catheter makes central venous catheter-induced endocarditis bear pathophysiologic similarity to the experimental model of endocarditis in animals [12]. In our patient, the central venous catheter was located in the upper vena cava and mural endocarditis was in the left ventricle. Infections with *C. parapsilosis *are more frequently the cause of catheter-related infections than all other *Candida *species combined [13]. On the other hand, the role of *C. parapsilosis *as a nosocomial pathogen associated with invasive devices and parenteral nutrition has been emphasized [14]. Characteristics of *C. parapsilosis *that may relate to its increasing occurrence in nosocomial settings include frequent colonization of the subungual space and an ability to proliferate in glucose-containing solutions, with a resultant increase in adherence to synthetic materials.

Our patient was not medically fit for heart surgery and was successfully treated with 8 weeks of intravenous caspofungin, although voriconazole was added due to persistence of positive blood cultures. Caspofungin has MICs somewhat higher for *C. parapsilosis *and *C. guilliermondii*, although the clinical significance is unclear [15]. This might explain the persistence of positive blood cultures 48 hours after the beginning of treatment with caspofungin. Ten previous cases of *Candida *endocarditis treated with caspofungin have been reported [16,17], four of which cured without cardiac surgery [17-20].

In our patient, combination therapy with caspofungin and voriconazole was an alternative to caspofungin monotherapy because of the persistence of positive blood cultures and the presence of renal insufficiency. On the other hand, continued fungemia at 3 days after the onset of caspofungin therapy cannot be considered that caspofungin was a failure, but given the known effect of grater MIC for *C. parapsilosis*, voriconazole was added without waiting for a possible resolution of candidemia. Voriconazole is a new triazole antifungal, while caspofungin is the first echinocandin antifungal. Voriconazole acts by inhibiting the synthesis of ergosterol in the fungal cell membrane. Caspofungin inhibits beta-1,3-D-glucan synthesis in the cell wall, a target present in fungal cells, and has shown excellent activity against *Candida *biofilm [21] as opposed to poor penetration of voriconazole [22,23]. Both agents are broad-spectrum, with efficacy against invasive *Aspergillus *and *Candida *infections. The efficacy of the antifungal combination therapy with caspofungin and voriconazole has been documented in a few case reports of invasive aspergillosis in patients with hematologic malignancies [24,25] and in a small series of patients with endogenous *Candida *endophthalmitis [26]. To our knowledge, the present report is the first description of successful treatment of *C. parapsilosis *mural endocarditis with caspofungin and voriconazole. Although relapsing endocarditis seems unlikely, the patient has been followed for less than 2 years and late relapse is still possible.

## Conclusion

In this paper, we firstly reported a non-operated patient with mural endocarditis by *C. parapsilosis *cured with a combination of caspofungin and voriconazole. The combination of these potent antifungal agents opens new perspectives in the management of severely ill patients with invasive fungal infection.

## Abbreviations

CT: computed tomography

MIC: minimal inhibitory concentration

SPSS: Statistical Package for the Social Sciences

## Competing interests

The author(s) declare that they have no competing interests.  C.S-R. is an employee of Merck Sharp &  Dohme de España, S.A.

## Authors' contributions

V. López-Ciudad, M.J. Castro-Orjales, M.J. de la Torre-Fernábdez, A. Pérez de Juan-Romero, M.D. Collell-Llach, and M.D. Díaz-López participated actively in the diagnosis, care, and follow-up of the patient.

V. López-Ciudad and M.J. Castro-Orjales obtained consent from the patient for this care report, reviewed the literature, provided clinical details, and drafted the manuscript.

V. León, reviewed the literature and drafted the manuscript.

C. Sanz-Rodríguez, reviewed the literature and gave helpful comments regarding the scientific content of the manuscript.

All authors read and approved the final manuscript.

## Pre-publication history

The pre-publication history for this paper can be accessed here:


